# Sources, Migration, Transformation, and Environmental Effects of Organic Carbon in Eutrophic Lakes: A Critical Review

**DOI:** 10.3390/ijerph20010860

**Published:** 2023-01-03

**Authors:** Xiaoguang Xu, Chao Wu, Dongyu Xie, Jie Ma

**Affiliations:** 1 School of Environment, Nanjing Normal University, Nanjing 210023, China; 2 Nanjing Institute of Environmental Sciences, Ministry of Ecology and Environment, Nanjing 210042, China

**Keywords:** lakes, eutrophic, organic carbon, source, migration and transformation, environmental effect

## Abstract

Organic carbon (OC) plays a leading role in the carbon cycle of lakes and is crucial to carbon balances at regional and even global scales. In eutrophic lakes, in addition to external river inputs, the decomposition of endogenous grass and algae is a major source of organic carbon. Outbreaks of algal blooms (algal eutrophication) and the rapid growth of aquatic grasses (grass eutrophication) can lead to the accumulation and decay of large amounts of algae and aquatic grass debris, which increases the intensity of the carbon cycle of lakes and greatly impacts aquatic environments and ecosystems. The structures, decomposition processes, and distribution characteristics of algae and higher aquatic plant debris in eutrophic lakes are different from mesotrophic and oligotrophic lakes. Studying their accumulation dynamics and driving mechanisms is key to further understanding lake carbon cycles and their many interdependent pathways. This paper focuses on the carbon sources, tracing technologies, migration and transformation processes, and environmental effects of OC in eutrophic lakes. Based on the existing knowledge, we further combed the literature to identify the most important knowledge gaps preventing an in-depth understanding of the processes and driving mechanisms of the organic carbon cycle in eutrophic lakes.

## 1. Introduction

Lakes represent interfaces at which the various spheres of the earth’s surface systems can interact [1], and they are important channels through which terrestrial carbon is respired and emitted to the atmosphere. The estimated global annual carbon dioxide emissions of lakes is about 0.14 PgC [2]. This means the lake carbon cycle is an important part of the global carbon cycle [3] and represents a large global carbon source/sink that will influence climate change [4,5]. In recent years, with increasing eutrophication of lakes, greenhouse gas emission from lakes has been intensifying [6,7]. In addition, in lake ecosystems, which are important hosts of nutrient cycling and energy flow processes, carbon cycling can vary significantly in time and space and among different lakes due to the photosynthesis and respiration of primary producers, microbial methanogenesis, methane oxidation, and other processes [8,9]. Therefore, carbon cycling and the related lake biogeochemical processes must be studied to better understand their roles in global systems.

Organic carbon (OC) is a key component of the lake carbon cycle. Its migration and transformation are core links within the lake ecosystem carbon cycle that directly affect the storage status of the lake carbon pool [10]. Generally, under anaerobic conditions OC is mineralized and degraded to produce CH_4_, while under aerobic conditions OC can be converted to CO_2_ through the aerobic respiration of microorganisms or photochemical reactions [11]. The scale and stability of a lake OC reservoirs depend on the dynamic balance between OC input and output. Recent observations have noted that fresh carbon (i.e., unstable and easily decomposable carbon) sources can have large impacts on a lake’s ecosystem, either promoting or inhibiting the metabolic rates of OC, which can greatly impact the lake carbon pool [12]. At present, the ecological problems that are common in lakes in China and globally, such as eutrophication, black and odorous water, and greenhouse gas emissions, are rooted in the fact that large amounts of biogenic carbon have altered the source–sink relationship of lake carbon pools, resulting in imbalances in the carbon budgets of lake ecosystems [13,14,15,16].

Lakes are often closely associated with different types of aquatic ecosystems, such as wetlands, estuaries, and groundwater. As such, as lake carbon budgets are transformed, large amounts of OC and various microorganisms from different environments are constantly mixing and interacting at the air–water–sediment interface [4]. Due to the relatively small ecological environment loads of lakes, their carbon balance systems are highly sensitive to changes in the OC content of internal and external sources [17,18]. The interactions between OC and microorganisms are affected by OC concentrations, chemical compositions, and environmental factors [19,20]. At present, the temporal and spatial distributions, bioavailability, and key processes controlling OC in lakes remain unclear. At the same time, climate warming has played a key role by accelerating the generation of algal OC and increasing microbial activity, making it more difficult to predict how biogeochemical processes will be affected by OC from various sources in eutrophic lakes [21,22,23,24]. Therefore, to consolidate our knowledge and provide insight into the processes and mechanisms controlling the interactions among organic carbon and the cycling of other elements in eutrophic lakes, this paper presents a review of the carbon sources, tracer techniques, transport and transformation processes, and environmental effects of OC in eutrophic lakes to facilitate the further exploration of the scientific issues involved.

## 2. OC Source Analysis

### 2.1. Oc Sources

**Figure 1:**
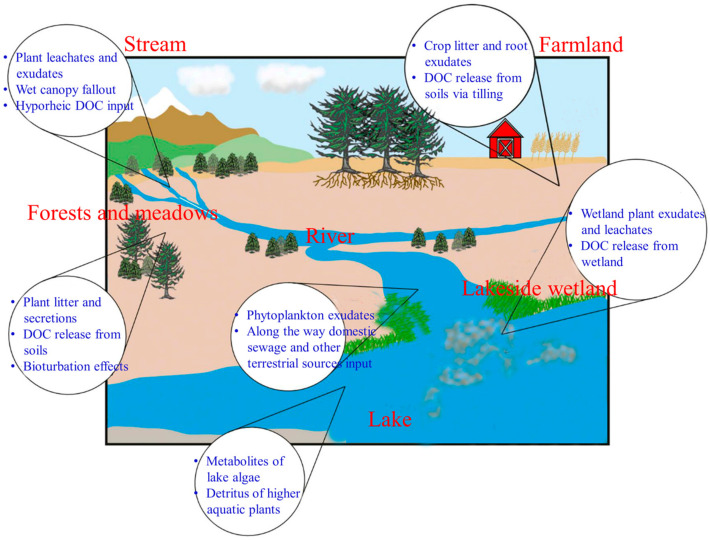
Potential sources of OC in the “staggered zone” of aquatic ecosystems [39].

In lake ecosystems, OC is mainly composed of dissolved organic carbon (DOC) and particulate organic carbon (POC). DOC refers to the OC with particle sizes less than 0.45 μm and POC refers to the OC with particle sizes greater than 0.45 μm [25]. In broad terms, the sources of lake OC can be categorized as exogenous or endogenous (Figure 1). Exogenous OC mainly comes from soil erosion, terrestrial plant debris, root exudates, production, and domestic wastewater carried by rain water and rivers into the lakes [26,27]; endogenous OC is closely related to biological activities and mainly comes from the residues of large aquatic plants (floating plants, emergent plants, hygrophytes, and submerged plants), algal debris, and products of their sedimentation and decomposition [28]. In terms of chemical composition, OC contains large amounts of various sugars, proteins, fatty acids, lignin, tannins, celluloses, and humus.

In recent years, under the dual influences of natural processes (rainstorm, flood, typhoon, etc.) and human activities (sewage discharge, etc.), the proportion of lake OC contributed by exogenous sources has gradually increased. There are obvious seasonal and regional differences in the sources of OC in lakes around the world. On the one hand, it is affected by basin environment (such as basin area, basin topography, plant primary productivity, hydrodynamic process, precipitation intensity, etc.). On the other hand, it is affected by human activities (such as land use type, tillage intensity, fertilization, damming, water storage, wastewater discharge, etc.). When people unilaterally pursue economic development and constantly increase the intensity of land cultivation, wastewater discharge and fertilizer application, the proportion of exogenous OC input in lakes will continue to increase, which will further destroy the lake carbon balance system. Photosynthetic intensity, biological activity (algae activity, etc.), microbial degradation, photodegradation, metal ion complexation, and salinity in the aquatic ecosystem can all affect the concentration of DOC in the water [25]. The redox conditions, microbial activity, and pH of the sediment are also important factors affecting the distributions of OC [29]. Carpenter et al. showed that DOC in oligotrophic lakes was mainly imported from exogenous sources [30]. Farjalla et al. found that the DOC in Batata Lake in the Amazon River basin was mainly imported from exogenous sources during high water levels and mainly sourced from endogenous sources during low water levels [31]. The δ^13^CDOM value of Kasumigaura Lake in Japan was higher in spring than in autumn, and the main source of DOC was endogenous during an algae outbreak in spring [32]. The OC of Taihu Lake in China was mainly exogenous, and the contribution of algal-derived carbon to total OC in sediments was low (17.0%~20.2%) [33]. Ye et al. analyzed the sources of POC in the upper waters of some lake areas of Taihu Lake using carbon stable carbon isotopes and found that algae were the main source of POC in the lake’s surface waters [34], while the POC in the surface waters of Chaohu Lake, Poyang Lake and Hongze Lake was mainly derived from exogenous inputs [35]. Generally, exogenous carbon is the main driving force of lake carbon cycles [36,37]. The exogenous carbon will undergo various biological and physical processes and most of it sinks to the lake bottom as sediment. Differences in POC content can be used to indicate input sources and primary productivity of OC in freshwater ecosystems. Exogenous and endogenous OC have different functions and roles in material circulation and energy flow processes in lakes, and jointly affect the carbon deposition rate, biological production, and mineralization efficiency of OC [38].

### 2.2. OC Tracing Technology

OC content can be used to characterize the organic matter inputs and productivity in lakes [40]. OC from different sources has great impacts on the carbon deposition rates and mineralization efficiencies of lakes [8,41]. Therefore, it is of great importance to understand the lake carbon cycle, which can be achieved by tracing the dynamic changes in OC from different sources along the pathway from the aqueous phase to sediment, during sediment carbon burial, and through mineralization processes.

#### 2.2.1. Carbon Stable Isotope

Based on the variations in carbon stable isotope, the contributions of different sources of OC in the water and sediment of lake ecosystems can be effectively tracked. At present, relatively accurate environmental data can be obtained using carbon stable isotopes, which have been widely used in the study of various types of lakes [42,43,44,45,46]. Stable isotope data analysis methods have also evolved and diversified over time as statistical methods have advanced.

In eutrophic lakes, many studies have used the algae isotope-labeling method to trace the migration and transformation of algae-derived carbon in the ecosystem, including the contribution of lake algae to the carbon pool of the food web [47], the impact of the biodegradation of algae-derived carbon on the DOC composition in water [48], and the migration and transformation of algae-derived carbon as it moves to higher trophic levels, e.g., zooplankton [49,50]. These studies have provided important references in the study of carbon cycling in eutrophic lakes using isotope-labeling technology. It is necessary to further develop the estimation of the contribution rates of algal-derived carbon to different forms and components of water and sediment facies at the different stages of the algae bloom decline process using an isotope-labeling technique based on isotope end-member mixed model. How to use the carbon stable isotope-labeling technique to trace the migration, transformation, distribution, and turnover rate of algal-derived carbon in the sediment–water–air phase during the algae bloom decline process is an important field for further study in the future.

#### 2.2.2. Biomarker Method

Lipid compounds, including n-alkanes, fatty acids, and alcohols, are widely used in OC source identification studies and can be obtained from sediments by organic solvent extraction [51].

##### N-alkanes Tracing

Indicators such as the characteristics of the peak cluster distribution and the carbon dominance indexes of n-alkanes with different carbon chain structures can indicate the relative contributions of different sources to OC. Compared with atmospheric samples, n-alkanes in lake sediments are well preserved, have a good resistance to microorganisms and degradation, are easy to collect and preserve during sampling, and contain much information, making them suitable for tracing the migration and transformation of OC in lake ecosystems [52]. Furthermore, a recently developed stable carbon isotope technique for monomeric n-alkanes allows researchers to precisely distinguish the biological sources of n-alkanes by the differences in the stable carbon isotope ratios of monomeric n-alkanes [53].

##### Fatty Acid Tracing

Fatty acids are a major organic compound contained in living organisms, and are produced mainly by bacteria, algae, higher plants, etc. Due to their structural diversity, source specificity, and relative stability, fatty acids are suitable for tracking the migration and transformation characteristics of OC from different sources in the ecosystem. Sources can be effectively traced according to differences in the lengths of fatty acid carbon chains, number of double bonds, double bond positions, and conformation of OC from different sources.

#### 2.2.3. Three-Dimensional Fluorescence Spectroscopy (3D-EEMs)

3D-EEMs can be used to conduct semi-quantitative analyses of dissolved organic matter (DOM) [54]. This method quickly and comprehensively identifies DOM components using fluorescence information by characterizing fluorescence intensity, excitation wavelength, and emission wavelength variation. 3D-EEMs has various advantages over other tools: it is convenient to operate, highly sensitivity, and does not easily destroy the organic composition of samples [55]. 3D-EEMs data are most often analyzed using the peak value method, regional integration method, and parallel factor analysis (PARAFAC). Among them, PARAFAC is best suited to identifying the components of 3D-EEMs and solving problems caused by multiple overlapping fluorescence peaks [56]. Although 3D-EEMs has been widely used in DOM research, it has difficulty detecting non-fluorescent components in DOM and analyzing samples at the molecular level, which limits its usefulness in OC studies.

#### 2.2.4. Fourier Transform Ion Cyclotron Resonance Mass Spectrometry (FT-ICR MS)

At present, FT-ICR MS is capable of obtaining the highest resolution mass spectrum, which can be used to accurately analyze DOM composition at the molecular level [57,58,59,60]. A large number of scholars have used FT-ICR MS to resolve the compositions of lake DOM, which has greatly improved our understanding of the structural characteristics of lake OC [61]. FI-ICR MS has significant advantages for characterizing the composition of DOM, such as high sensitivity, good selectivity, and the ability to detect the responses of thousands of compounds to excited substances. However, this method requires complex experimental techniques and the characterization results can be affected by DOM enrichment extraction and ionization methods [62].

## 3. OC Migration and Transformation

### 3.1. Migration and Transformation Processes of Oc at Different Phase Interfaces

#### 3.1.1. Aqueous Phase–Gas Phase Interface

CO_2_ and CH_4_ in the atmosphere can enter lake water through dissolution, thus exchanging carbon with the lake water [4]. In nature, aquatic plants in lakes can absorb and fix CO_2_ from the atmosphere and water through photosynthesis and use it to synthesize their own OC, much of which will eventually leave the plant body and enter the litter carbon pool [63]. The OC in litter represents an endogenous source of lake OC and can eventually be dispersed in water as DOC and POC. In addition, phytoplankton residues and terrestrial pollutants transported into lakes through surface runoff, erosion, and leaching will release DOC and POC and further increase lake OC content. Generally, CO_2_ and CH_4_ can easily be formed from DOC and released into the atmosphere through bacterial respiration and mineralization of organic matter, while POC is more recalcitrant. In lake water columns, most organic debris settles to the bottom where it undergoes carbon cycling processes in the sediment.

#### 3.1.2. Aqueous Phase–Sediment Interface

**Figure 2:**
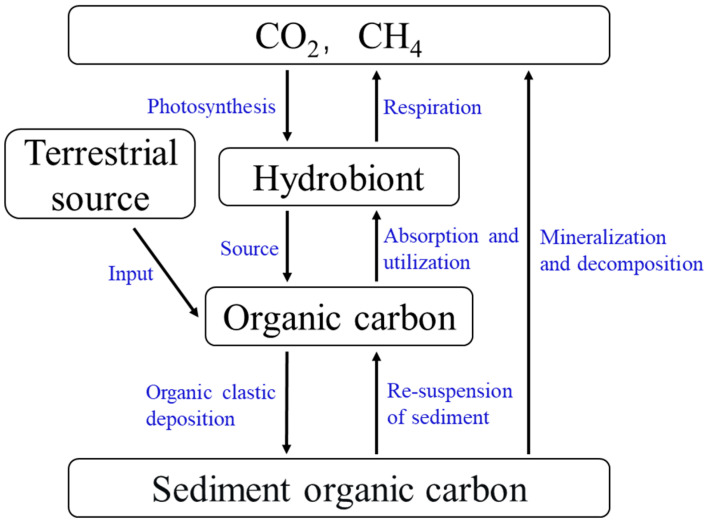
Diagram of the OC circulation pathways in lakes.

Sediment is an important part of lake ecosystems and an important storage reservoir of carbon. The high biological productivity of lakes drives the rapid deposition of carbon from biogenic sources, resulting in active carbon fixation and release at the interface between the aqueous phase and sediment (Figure 2). This transfer of OC is mainly from the degradation and deposition of dead aquatic organisms or the deposition of insoluble POC from the water column. The decomposition of OC in sediments releases inorganic carbon as CO_2_ and CH_4_, which is transferred into the overlying water column. In addition, OC in sediment may also be resuspended into the water column by water turbulence [29]. This is especially important in shallow lakes where disturbance by wind waves and lake currents can intensify the resuspension of sediments, which increases the release of OC from sediments to the water column.

### 3.2. Migration and Transformation Characteristics of Algae-Derived OC

**Figure 3:**
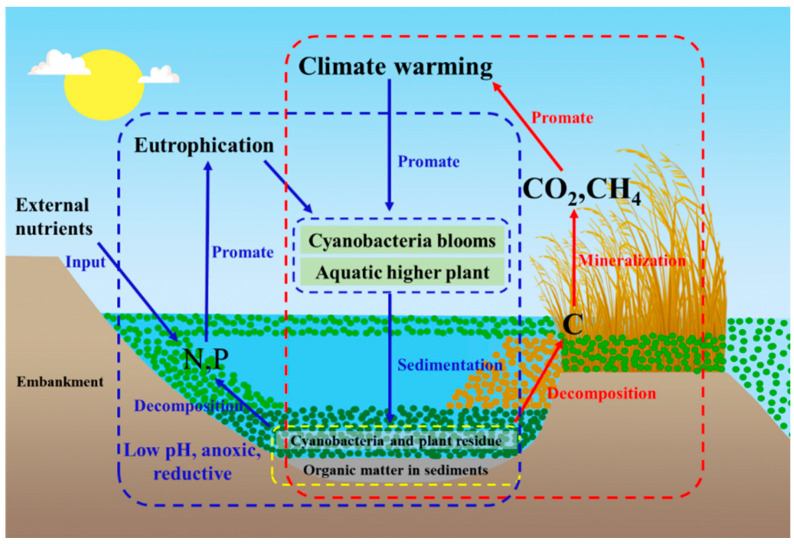
OC transport and transformation pathways in eutrophic lakes (blue arrows represent cycles affecting eutrophication, red arrows represent cycles affecting climate warming).

Algal bloom outbreaks are a significant feature of eutrophic lakes. With the elevated input of algal debris as blooms die, and the algal-derived OC can undergo degradation or heterotrophic utilization, or be transferred, accumulated, or exported (Figure 3). Algae decay consumes oxygen and releases algal toxins, which can cause the death of aquatic organisms due to hypoxia or ingestion of excess toxic substances [64,65]. When fish, shellfish, and other aquatic organisms die, their remains will also become an internal source of organic carbon.

In eutrophic lakes, because turbid lake water limits photochemical reactions, microbial mineralization is the main OC degradation process [66]. Algal-derived POC is degraded and mineralized by microorganisms. Under aerobic conditions, algal-derived POC acts as an electron donor and is decomposed to produce CO_2_ and H_2_O using O_2_, SO_4_^2−^, Mn^4+^, and Fe^3+^ as electron acceptors [67,68]; under anaerobic conditions, algal POC generates CO_2_ and CH_4_ through the metabolism of CH_4_ producing microorganisms, whose metabolic pathway involves both acetate fermentation and CO_2_ reduction [68].

Influenced by meteorological factors and hydraulic conditions, a large amount of algal debris usually accumulates in localized areas of large lakes, such as lake bays, lakeside zones, erosion chambers, and emergent plant clusters, forming active particulate organic carbon pools [14,69]. A large number of scholars have studied the changes in organic carbon during the decay and accumulation processes of algal blooms, including changes in DOC content in the aqueous phase [70,71] and changes in the composition of colored soluble organic matter (CDOM) [72,73]. These studies revealed the influence of algal bloom-derived degraded carbon on the distributions of different carbon morphological compounds in the aqueous phase. However, systematic studies on the carbon balance and flux at the sediment–water interface and their influencing mechanisms during the decay of algal blooms are lacking.

Algal-derived OC settles to lake bottom sediments, providing important food and energy sources for lake bottom organisms [50]. Some of the more reactive OC compounds are preferentially degraded, including pigments, sugars, proteins, and amino acids, and eventually refractory OC accumulates in the sediment in the form of humic substances [42]. Therefore, the deposition of algal OC in lake sediments and its subsequent degradation directly affect carbon burial and the sediment carbon pool. The differences in the structural dynamics of the siltation layers between lakes with rapid deposition due to eutrophication and lakes with slow natural deposition are still unclear. Therefore, it is important to understand how the unique reducing environment of the thicker algal remnants formed by rapid siltation affects the migration and transformation processes of organic carbon. Furthermore, whether this environment accelerates the succession of lake ecosystems from eutrophic environments to humic and marshy environments needs to be further explored.

### 3.3. Driving Mechanisms

Under eutrophic conditions in lakes, the microbial community structure will differ from the natural state, so the products and rates of the decomposition of organic matter from different sources (algal/herbaceous sources) will also differ. Furthermore, many physical factors will affect the microbial communities and migration and transformation of organic carbon in eutrophic lakes, including temperature, DO, pH, input quantities of organic carbon, microorganisms in water, and soil and hydrodynamic conditions. Therefore, changes in environmental conditions will affect the decomposition of organic matter directly as well as indirectly through relationships with key microbial communities. Generally, in regard to climate warming and eutrophication, temperature and high algal inputs are the factors that most strongly influence degradation processes.

During eutrophication in lakes, large amounts of algal residues are transferred into the sediment–water system, which significantly increases the organic carbon content of the lake and increases the lake sediment’s role as a “carbon sink”. Algae have simple compositions and fast decomposition speeds, and up to 50% of their dry weight can be decomposed within 4 days [74], whereas aquatic plants are highly fibrous and decompose relatively slowly, usually taking 0.5–12 months to decompose completely [75]. In systems with sediment composed of mixed algal–plant debris, the preferential decomposition of algal-derived carbon by bacteria can have an effect on the decomposition of plant carbon. It has been shown that the rapid metabolism of easily decomposed organic matter can be associated with increases in the decomposition rate of refractory organic matter, revealing a co-metabolism effect (co-decomposition process, co-metabolism effect) [76]. For example, Zhang et al. studied the microbial degradation of volatile organic compounds in toluene-acclimated sludge and found that, in addition to effectively degrading toluene, there was a good chlorobenzene degradation rate [77]. However, Dai et al. studied the metabolic processes of organic matter composed of different types of plants in a coastal estuarine zone and found that when mixed diatoms (easily decomposable) and intercalated rice grasses (refractory) were degraded simultaneously, both of their degradation rates were negatively affected, showing a negative co-degradation effect [78]. Canfield proposed the pseudo-G model to analyze the interrelationships between the degradation processes of easily decomposable and recalcitrant organics [79]. Therefore, to understand mixed algae–plant decomposition and how their combinations affect the migration and transformation of organic carbon in sediments, it is necessary to investigate the co-metabolism effects between different combinations of carbon sources.

Co-metabolism relationships are further complicated by temperature, which can also significantly affect the migration and transformation of organic carbon in sediments. In northern latitudes, cold climate conditions can facilitate the storage of organic carbon in lakes [80], while warm inland lakes exhibit strong organic carbon degradation, resulting in large amounts of carbon being released into the atmosphere [81]. This is because the biological activity of microorganisms is highest and the organic matter degradation rates are fastest when temperatures are near the optimum temperature for bacterial growth [82]. O’Reilly’s survey of hundreds of lakes around the world showed that lakes around the world are warming faster than oceans and the surrounding air, which will have a general impact on the carbon cycling processes of lakes [83]. Therefore, temperature should be considered the main factor controlling the mineralization of organic carbon in the sediments of inland water bodies such as lakes. However, it is unclear whether the sediments from different organic sources have different sensitivities to temperature changes, and how these differences will affect the migration and transformation of organic carbon.

#### 3.3.1. Microbial Processes

Microorganisms are an important part of the lake ecosystem and the main driver cycling carbon elements and other substances in the water. In addition, floods caused by typhoons also play an important role in the migration and transformation of OC. Heavy rainfall events can significantly increase the proportion of terrigenous OC input within a short period of time, resulting in the accumulation of a large amount of highly unstable organic matter in the basin [84]. It provides an excellent substrate for heterotrophic respiration, thus affecting the OC cycle of lakes [85]. Indeed, the migration and transformation of OC in lakes are mainly dependent on bacteria and other microorganisms [1]. The bioavailability of different sources of OC to bacteria and other microorganisms varies somewhat among microbial species. In order to account for the biological availability of OC types and their structural differences, OC can be divided into stable OC and unstable OC [86]. Stable OC has relatively complex chemical structures and low bioavailability, while unstable OC has relatively simple structures and can readily be used by bacteria [86]. In nature, OC is rarely composed of one type or from a single source and the decomposition process is rarely undertaken by any one microorganism. Therefore, co-metabolism processes are key to carbon cycling in lakes.

**Figure 4:**
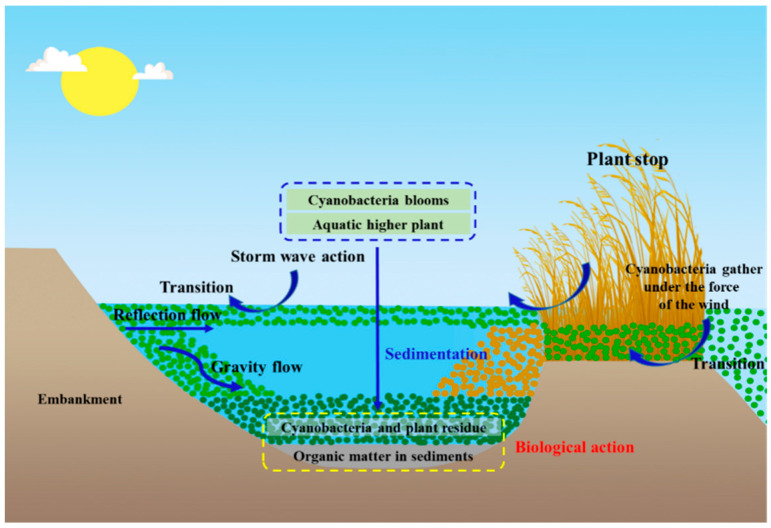
Schematic diagram of algal accumulation pathways in a typical lakeshore erosion channel.

Lake ecosystem co-metabolism occurs when multiple sources of organic matter, such as algae and higher aquatic plant residues, accumulate and mix in the same space where they are decomposed, mainly occurring in areas where multiple OC sources converge [87,88]. In general, algal blooms in eutrophic lakes are often affected by wind and accumulate in nearshore zones, or rapidly accumulate and deposit in localized areas such as those with concentrations of aquatic plant communities and erosion canals (Figure 4). The huge biomass of algal blooms will aggregate at the water surface before settling to the bottom where it mixes with higher plant residues, exogenous organic matter, and sediment organic matter to form a mixed accumulation layer. The upper layer of the accumulation layer is generally the newly accumulated material from algal blooms and aquatic plant residues, and the next layer is generally algal grass residues and sediment deposited in previous years. These algal debris and higher aquatic plant residues continue to accumulate over time, and because their organic components are very extensive and complex the degree of decomposition varies greatly, providing variable material inputs for the occurrence of lake co-metabolism.

**Figure 5:**
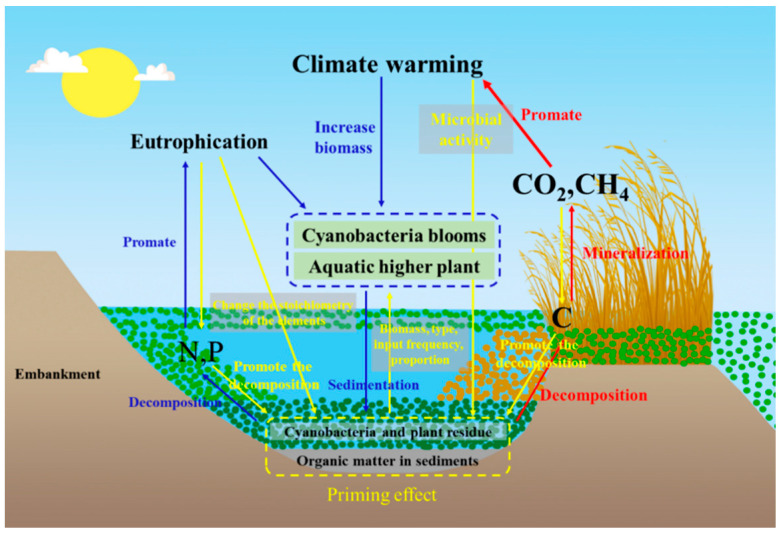
Co-metabolic pathways in eutrophic lakes and their potential influencing factors.

The co-metabolic processes of multi-source organic detritus occur first in the surface layers of sediments. Compared with a single organic clast, the catabolic processes of mixed multi-source organic clasts will be significantly different. The co-metabolism of algal-derived organic matter (relatively easy to decompose) and higher aquatic plant residues (relatively difficult to decompose) in the surface sediments, combined with the co-metabolism of fresh algal grass residues (relatively easy to decompose) and undissolved plant residues and humus (relatively difficult to decompose) in sediments further accelerate the decomposition processes of lake OC (Figure 5). Under a background of continuous global warming, the increased frequency of algal bloom outbreaks will also further affect the carbon cycling in eutrophic lakes.

#### 3.3.2. Photodegradation Mechanisms

For a long time, research on OC degradation by light focused mainly on arid and semi-arid terrestrial ecosystems [89,90], with little research in aquatic ecosystems. In aquatic ecosystems, the decomposition of OC is generally attributed to microbial action, while photodegradation is often ignored [91]. Recently, more and more scholars have observed that photoperiod can accelerate OC decomposition in water [92]. However, the photochemical characteristics and photodegradation mechanisms of OC in lake ecosystems are very complex, and are mainly affected by the structural characteristics of the OC and environmental conditions [93]. Generally speaking, the photodegradation of OC can be divided into direct photodegradation and indirect photodegradation. Through direct photodegradation, OC in lakes can be decomposed into CH_4_ and CO_2_ and released into the water [94]. In indirect photodegradation, light radiation acts on photosensitive substances (lignin, humus, CDOM, etc.) to produce reactive oxygen species that participate in the process of lake OC cycling [95,96].

Photodegradation of OC usually causes two types of changes, photobleaching and photomineralization. The photochemical degradation of macromolecular organics such as lignin and α-cellulose can be promoted when the amount of ultraviolet (UV-B) radiation contained in sunlight increases, and the availability of microorganisms to OC can be improved, thus accelerating the decomposition of OC [97]. Photobleaching refers to the phenomenon where the absorbance decreases after the chromophores in OC absorb ultraviolet light [98]. Photobleaching can increase the transparency and improve the optical properties of the water column, and thus have an important impact on photosynthesis by phytoplankton and submerged plants. Photomineralization refers to the degradation of OC by light into small inorganic molecules (CO_2_, CH_4_, etc.), thus affecting the OC circulation rate and flux [99]. Under solar irradiation, in addition to direct photodegradation, OC can undergo indirect photodegradation wherein a variety of active substances (reactive oxygen species, etc.) are produced [100,101].

## 4. Environmental Effects of OC

In the water columns of eutrophic lakes, large amounts of algae and aquatic grass debris accumulate and decay, significantly changing the intensity of the lake carbon cycle, while also having a large impact on the water environment and aquatic ecosystem. These changes can intensify the formation of the anaerobic environment in water columns, accelerate the release of nitrogen and phosphorus, promote the emission of odorants, promote the emissions of greenhouse gases, and lead to lake flooding or black water masses.

### 4.1. Eutrophication Aggravates the Formation of Anaerobic Environments in Water Columns

The high content of algal-derived organic matter and the related decomposition and oxygen consumption can lead to anoxic conditions in water [102,103,104]. Zhu et al. studied the effects of cyanobacteria bloom decay on sediment nitrogen and phosphorus release in Taihu Lake using indoor simulation experiments and observed that the massive die off of cyanobacteria formed an extremely anoxic environment in the water column, and the associated increase in aqueous phase nutrient salts was significant compared to the degree of anoxia and the duration of the cyanobacterial bloom [105]. Ma found that the mixture of algae, aquatic grass residues, and sediment will have a positive co-metabolism effect in water, resulting in a sharp drop in the concentration of dissolved oxygen and redox potential, which were more pronounced with mixed OC sources compared to when an individual OC source was decomposed [106]. In addition, in some local areas, anaerobic waters will cause extreme water quality deterioration events such as “black water masses” and “lake flooding”, causing great risks to aquatic ecological environments [107,108].

### 4.2. Eutrophication Aggravates the Release of Nitrogen and Phosphorus Elements

The decomposition of algal-derived organic matter releases large amounts of nutrient salts, some of which are deposited in the sediment while some stay in the water body and provide nutrients for the growth of algae and aquatic plants [109]. At present, studies on the release of inorganic nutrients such as C, N, and P during the decline of a cyanobacteria bloom have mainly focused on the changes in the nutrient concentrations in lakes [110], the mechanism of microbial action [111], and the mechanisms coupling nutrient release and aquatic plants [112]. Li et al. showed that the release rate of phosphorus was higher than that of nitrogen during the bloom decline of cyanobacteria, but the increase in the dissolved total nitrogen concentration in water caused by it lasted longer than that of the dissolved total phosphorus [74]. Ma showed that the co-metabolism of algal residues that settle to the sediment surface resulted in a significant increase in sediment TOC, TN, and TP concentrations [106].

### 4.3. Eutrophication Promotes the Emission of Odoriferous Substances

The massive accumulation of algae-derived organic matter through siltation can lead to the proliferation of sulfate-reducing bacteria that release methionine during the decomposition of algae, which rapidly decomposes into thioether-like odorous compounds [113,114]. The release of large amounts of volatile organic carbon compounds (VOCs) leads to odors from lake waters. Among them, dimethyl sulfide (DMS), dimethyl disulfide (DMDS), and dimethyl disulfide (DMTS) are important substances that cause strong irritating odors when “lake flooding” occurs [115]. One such water crisis event occurred in Wuxi, China in 2007 when the concentrations of DMS, DMDS, and DMTS in the lake water were particularly high [116]. However, while excessive amounts of VOCs can be detrimental, the transformation of algal-derived POC to VOCs is an important part of the carbon cycle in eutrophic lakes.

### 4.4. Eutrophication Enhances Greenhouse Gas Emissions

The mechanisms by which CO_2_ and CH_4_ are emitted when cyanobacteria bloom, accumulate, die, and are decomposed in eutrophic lakes has attracted the attention of scholars both in China and internationally. Studies mainly include the impacts of accumulated cyanobacteria biomass on greenhouse gas flux production [117], the role of methanogenic bacteria in CH_4_ production during cyanobacteria decomposition [118,119], the comparison of methane production rates of cyanobacteria bloom and macro-aquatic plant source OC decomposition [120], the correlations between temperature change and CO_2_ and CH_4_ fluxes from eutrophic lakes [121,122], and the effect of CH_4_ release during cyanobacterial decomposition on the lake carbon cycle with and without cyanobacteria [123]. Many studies have observed that the decomposition of algae-derived carbon as cyanobacteria blooms decline increases the release intensity of CO_2_ and CH_4_ from lakes. For example, when the southeast winds prevail in summer in Taihu Lake, which is a typical eutrophic lake, the cyanobacteria accumulate in Zhushan Bay and the western coastal zone where they are captured and stacked by aquatic plants such as reeds, forming a thick cyanobacteria accumulation layer that promotes the production of greenhouse gases as it decays [124]. Yan et al. found that the concentrations and saturation states of CH_4_ and N_2_O greenhouse gases in the water of the cyanobacteria accumulation area near Taihu Lake were higher than those in the open lake area [125].

### 4.5. Eutrophication Enhances “Lake Flooding” and “Black Water Mass”

A survey of carbon cycle imbalances in localized areas of eutrophic lakes showed that, in lakes such as Taihu Lake and Hongze Lake in China, the thickness of algal residue siltation along lakeshore banks or in lake bays is often up to about 1 m. This rapid siltation of residues in the surface sediments leads to the uplifting of the lake bottom and accelerates the development of lake marshes [126,127].

## 5. Outlook on Eutrophication and Carbon Cycling Research

In recent years, against the background of the ever-strengthening influence of human activities on lake ecosystems, governments and scholars worldwide have focused on eutrophic lakes and climate warming, and much progress has been made in understanding the OC cycles of eutrophic lakes. However, the sources of OC in lakes continue to become more and more complex. For example, eutrophication in shallow lakes can result in sediments that are generally algal accumulation dominated, mixed grass–algae accumulation dominated, or grass-type accumulation dominated, with a wide range of algal and aquatic grass species contributing to the biomass. The underlying driving mechanisms also continue to increase in complexity, and further research on the OC cycling process in eutrophic lakes is required. To better understand OC cycling in eutrophic lakes, it is important that we focus on the following aspects: (1) New characterization techniques need to be developed for OC source analysis. At present, no single method is sufficient, and it is usually necessary to combine several methods or indicators to compare and corroborate each other, as well as to expand and revise them, in order to distinguish different OC sources comprehensively and precisely. (2) The degradation mechanisms of OC are still unclear, especially the mixed decomposition processes of OC from multiple sources in eutrophic lakes. It is necessary to further study the driving mechanisms and influencing factors of microbial processes (co-metabolism effects, etc.) and physicochemical processes (photodegradation, etc.). (3) It is necessary to more accurately evaluate the environmental effects that OC has and its possible promoting or inhibiting effects on different processes. For example, in addition to focusing on the promoting effect of algae on greenhouse gases, it is also necessary to consider the inhibition effect of increasing water sulfur concentrations on methane emissions. (4) It is not sufficient to study the impact of the carbon cycle on the lake water environment as an isolated system; we must comprehensively consider the coupling effects of carbon element and nitrogen, sulfur, iron, phosphorus, and other substances and their impacts on regional water environments.

## Data Availability

Not applicable.
